# Physical Health Monitoring of Patients Prescribed Depot Antipsychotic Medication in North West Edinburgh Community Mental Health Team (CMHT)

**DOI:** 10.1192/bjo.2022.427

**Published:** 2022-06-20

**Authors:** Adrianna Klejnotowska, Robyn Bailey, Alexandra Thompson, Jakub Wojtowicz, Josh Haggart, Hamsi Evans, Hae-young Choi, Adam Mallis, Anna MacLeod, Douglas Murdie, Vikki Argent

**Affiliations:** 1 NHS Lothian, Edinburgh, United Kingdom; 2 University of Edinburgh, Edinburgh, United Kingdom

## Abstract

**Aims:**

To assess the effect of interventions in the physical health monitoring of patients prescribed depot antipsychotic medications. We hypothesised that compliance with monitoring would improve post-intervention. It is well recognised that patients with severe mental illness have a significantly reduced life expectancy. Depot antipsychotic medication increases the risk of cardiovascular disease, metabolic syndrome, stroke and type 2 diabetes. The SIGN guidelines recommend that all patients on antipsychotic medications should have annual physical health monitoring. Baseline data of patients on depot antipsychotic medication in North West (NW) Edinburgh CMHT in 2019 demonstrated that this was not being achieved. We sought to create interventions to improve compliance with physical health monitoring for patients on depot antipsychotic medication.

**Methods:**

Baseline data were collected in 2019 for all patients under NW Edinburgh CMHT receiving depot antipsychotic medication (60 patients). The data addressed 9 domains including smoking status, blood monitoring, BMI and physical monitoring.

Following the baseline data collection interventions were put in place to increase compliance with monitoring. These interventions included a physical health questionnaire and training of staff in the CMHT to perform phlebotomy and ECGs.

Following these interventions the data (74 patients) were re-audited in 2020 following the same domains.

After this initial re-audit a physical health monitoring clinic was implemented in order to specifically target this patient population. The data (66 patients) were then re-audited in 2021.

**Results:**

Baseline data identified that domains were reached between 8% (Lipid monitoring) and 51% (glucose monitoring). Following the initial interventions 77% of domains improved in compliance. Between the two periods, notable improvements were observed in the monitoring of Blood Pressure (9% to 37%), ECG (20% to 43%) and lipids (29% to 46%). There was however a decline in all domains between the 2020 and 2021 data, with 66% of domains still having improved compared to 2019 data.

**Conclusion:**

Overall, interventions have improved compliance with monitoring of physical health for patients on depot antipsychotic medications. It is likely that continuing effects of the COVID-19 pandemic contributed to the decline between the 2020 and 2021 data. As a result of this audit a weekly physical health monitoring clinic has been set up and once formally established it is hoped that compliance with physical health monitoring will continue to improve. Limitations include effects of COVID-19 pandemic, inconsistency in documentation and patient non-attendance to the monitoring clinic. We recommend further audit cycles, with additional interventions being implemented as identified.

